# Budget impact analysis of a digital monitoring platform for COPD

**DOI:** 10.1186/s12962-023-00443-x

**Published:** 2023-06-04

**Authors:** Timothy J. Inocencio, Kimberly L. Sterling, Sibel Sayiner, Michael E. Minshall, Leanne Kaye, Umur Hatipoğlu

**Affiliations:** 1 Open Health Evidence and Access, Parsippany, NJ USA; 2 ResMed Science Center, 9001 Spectrum Center Boulevard, San Diego, CA 92123 USA; 3 Propeller Health, San Francisco, CA USA; 4 Cleveland Clinic, Cleveland, OH USA

**Keywords:** Chronic obstructive pulmonary disease, Cost, Remote monitoring, Digital health technology, Budget impact

## Abstract

**Background:**

Chronic obstructive pulmonary disease (COPD) is a progressive debilitating condition with frequent exacerbations that have a high burden for patients and society. Digital tools may help to reduce the economic burden for patients and payers by improving outcomes. The Propeller platform is a digital self-management tool that facilitates passive monitoring of inhaler medication utilization, potentially assisting the healthcare team to identify patients at risk of a COPD exacerbation who may require further intervention. This study estimated the budget impact of Propeller from commercial payer and Medicare fee-for-service payer perspectives.

**Methods:**

An Excel-based model was used to estimate the budget impact of Propeller for COPD patients in commercial and Medicare population sizes of 5 million members. Data on prevalence, baseline healthcare resource utilization (HCRU), and baseline use of rescue and controller inhaler medications with unit costs (adjusted to 2020 US dollars) were obtained from peer-reviewed literature. Data on reductions in HCRU during Propeller usage were based on direct evidence. Estimates for costs of remote monitoring were obtained from publicly available information. All patients were assumed to have insurance claims related to ongoing remote monitoring.

**Results:**

The estimated number of annual eligible COPD patients for commercial and Medicare was 212,200 and 606,600, respectively. Propeller decreased costs by an estimated $2,475 (commercial) and $915 (Medicare) per enrolled patient. The greatest increase in expenditure was for remote monitoring related expenses. After accounting for estimated reductions in hospitalizations, emergency department visits and short-acting beta-agonist use, total net savings were approximately $1.60 and $1.70 per-member per-month for commercial and Medicare payers, respectively.

**Conclusion:**

Propeller is projected to be cost saving from both the commercial and Medicare payer perspectives.

**Supplementary Information:**

The online version contains supplementary material available at 10.1186/s12962-023-00443-x.

## Introduction

Chronic obstructive pulmonary disease (COPD) is a progressive debilitating disease with high morbidity and mortality, affecting 15.5 million patients and causing 150,000 deaths annually in the United States (US) [1–3]. Total COPD-attributable medical costs in the US were estimated at $32.1 billion in 2010 and projected to reach $49.0 billion in 2020 [4]. Patients with COPD are at high risk of exacerbations, with nearly 20% experiencing one or more costly COPD-related hospitalizations annually [5].

Recommended long-term pharmacological management of COPD typically consists of combinations of inhaled medications referred to as controller or maintenance medications, including a long-acting beta-agonist (LABA), a long-acting muscarinic antagonist (LAMA), with or without an inhaled corticosteroid (ICS) [6]. Short-acting beta-agonists (SABA) are commonly used to treat acute symptoms and are referred to as rescue or reliever medications. Frequent use of SABA may reflect greater COPD symptom burden and underlying disease severity [7, 8].

Propeller is a digital health solution that uses an electronic medication monitor to passively capture inhaler medication utilization including the date and time of inhaler use. Inhaler usage data are transmitted to a patient-facing smartphone application to provide feedback on medication use and receive controller medication reminders and education. With patient permission, inhaler usage data may also be transmitted to a secure web-based clinician portal for healthcare providers (HCPs) to support care management. Clinicians receive notifications for patients with increased short-acting beta-agonist (SABA) use and/or low adherence to maintenance treatment. This data is intended to support clinical decisions and enable early intervention as part of a care management program [9].

Data on real-time SABA utilization may help HCPs to identify patients with worsening symptoms who may be at risk of exacerbations and might benefit from preemptive intervention, including medication adjustments [10–12]. Studies with Propeller have demonstrated reductions in SABA use, emergency department (ED) visits and hospitalizations in patients with COPD [13, 14]. Reductions in healthcare resource utilization (HCRU) may potentially be explained by better medication adherence and exacerbation identification during electronic inhaler monitoring [15].

Given the high prevalence and costs of COPD, and the increasing trends towards reimbursement of telehealth and remote patient monitoring [16], it is important to assess the costs of novel digital health interventions such as Propeller for patients with COPD. This study estimated the budget impact of Propeller in COPD patients from both commercial and Medicare fee-for-service payer perspectives.

## Material and methods

### General overview and model assumptions

A Microsoft Excel-based model was created to estimate the 1- to 3-year budget impact of Propeller usage for patients with COPD from the commercial and Medicare payer perspectives. The model estimated the total number of patients with COPD, stratified by age and symptom burden (based on Global Initiative for Chronic Obstructive Lung Disease [GOLD] 2017 ABCD criteria) [17]. We adopted the GOLD 2017 ABCD clinical classification scheme, which grouped patients based on symptom burden and exacerbation risk. According to the GOLD 2017 ABCD classification, patients who have low symptom burden and low exacerbation risk belonged to Group A. Group B consisted of patients with lower exacerbation risk but high symptom burden. Both groups C and D included patients with high exacerbation risk but with low and high symptom burden, respectively. The model then compared scenarios with and without Propeller coverage while accounting for SABA and maintenance inhaler usage, HCRU, and estimated Propeller utilization over time. All model input parameters are provided in Table 1.

**Table 1:** Model input parameters

Parameter	Value	Source
Population parameters, proportion of patients		
Age distribution (commercial)		Current Population Survey, 2018 [19]
< 40 years	53.0%	
40–64 years	35.0%	
> 65 years	12.0%	
Age distribution (Medicare)		Current Population Survey, 2018 [19]
< 40 years	3.0%	
40–64 years	12.0%	
> 65 years	85.0%	
COPD prevalence		CDC [20]
40–64 years	7.6%	
≥ 65 years	13.2%	
COPD groups		Cabrera-Lopez et al. [21]
GOLD A	53.4%	
GOLD B	26.7%	
GOLD C	8.2%	
GOLD D	11.7%	

	GOLD A	GOLD B	GOLD C	GOLD D	
Healthcare resource use parameters by GOLD category, number per patient per year					
Annual medical resource use					Wallace et al. [5] and Bhatta et al. [22]
Outpatient visits	2.64	2.64	2.64	2.64	
ED visits	0.11	0.19	0.22	0.22	
Hospitalizations	0.36	0.46	0.56	0.56	
Readmissions	0.04	0.07	0.08	0.08	
Inhaled medication use parameters by GOLD category					
Weekly SABA utilization, puffs/week	5.81	8.82	5.81	12.78	Gondalia et al. [7]
Proportion of patients utilizing controller medications					Internal clinical expert opinion
LAMA monotherapy	40%	30%	0%	0%	
LABA monotherapy	20%	0%	0%	0%	
LABA + ICS	0%	30%	60%	40%	
LAMA + LABA	40%	40%	35%	10%	
LABA + LAMA + ICS	0%	0%	5%	50%	
PDC for controller medications	0.54	0.60	0.56	0.62	Wallace et al. [5]
Unit cost inputs, USD					
Medical resource unit costs (commercial)					Wallace et al. [5] and inflated to 2020 USD using the medical care component of the CPI [23]
Office/outpatient visits		126			
ED visits		3,065			
Hospitalizations		25,839			
Medical resource unit costs (Medicare)					Wallace et al. [5] and inflated to 2020 USD using the medical care component of the CPI (23) and converted to Medicare payments using commercial-to-Medicare payment ratios[24]
Office/outpatient visits		88			
ED visits		1,161			
Hospitalizations		13,671			

Parameter	Value	Source
SABA medications (WAC), USD		Red Book Online, 2020 (25)
Albuterol sulfate HFA	35.98	
Albuterol sulfate HFA (Cipla)	57.75	
Proair Digihaler	146.67	
Proair HFA	66.88	
Proair Respiclick	62.52	
Proventil HFA	79.73	
Ventolin HFA	55.36	
Atrovent HFA	332.70	
Combivent Respimat	426.45	
Albuterol/ipratropium	426.45	
Daily controller medication cost, USD		Red Book Online, 2020 (25)
LAMA	14.80	
LABA	10.40	
LABA + ICS	10.50	
LABA + LAMA	17.14	
LABA + LAMA + ICS	19.11	
Remote monitoring*		
Yearly frequency		Assumption
HCPCS Code 98975	1	
HCPCS Code 98976	6	
HCPCS Code 98980	6	
Fees, USD		CMS 2022 Physician Fee Schedule [18]
HCPCS Code 98975	19.38	
HCPCS Code 98976	55.72	
HCPCS Code 98980	50.18	
Reduction in HCRU with Propeller		Alshabani et al. [14] (base case)
ED visits	55%	
Hospitalizations	30%	
Reduction in SABA use with Propeller	59.4%	Chen et al. [13]

*For the purposes of this analysis, we conservatively assumed all patients in commercial plans were able to make a reimbursement claim to the respective payer

**HCPCS Code 98,975: Remote therapeutic monitoring (e.g. respiratory system status, musculoskeletal system status, therapy adherence, therapy response); initial set-up and patient education on use of equipment; HCPCS Code 98,976: Remote therapeutic monitoring (e.g. respiratory system status, musculoskeletal system status, therapy adherence, therapy response); device(s) supply with scheduled (e.g. daily) recording(s) and/or programmed alert(s) transmission to monitor respiratory system, each 30 days; HCPCS 98980: Remote therapeutic monitoring treatment, physician/other qualified health care professional time in a calendar month requiring at least one interactive communication with the patient/caregiver during the calendar month; first 20 min

CMS: Center for Medicare and Medicaid Services; COPD: chronic obstructive pulmonary disease; ED: emergency department; GOLD: Global Initiative for Chronic Obstructive Lung Disease; HCPCS: Healthcare Common Procedural Coding System; HCRU: healthcare resource utilization; ICS: inhaled corticosteroid; LABA: long-acting beta-agonist; LAMA: long-acting muscarinic antagonist; PDC: proportion of days covered; SABA: short-acting beta-agonist; WAC: wholesale acquisition cost

Key model assumptions were:

- Use of Propeller was accompanied by care management from the provider.
- Billing (insurance claims) for remote monitoring occurred for the entire patient population using the Centers for Medicare & Medicaid Services (CMS) Physician Fee Schedule [18].
- COPD symptom burden and risk of exacerbations were based on GOLD 2017 ABCD criteria [17] and (due to data limitations) did not differ between age groups.
- Data for HCRU differed by GOLD 2017 ABCD category.
- Based on published evidence, Propeller only reduced COPD-related hospitalizations, ED visits and rescue inhaler medication use.
- Due to data availability limitations, no effect on HCP office visits or non-ED outpatient visits was assumed, but these components of HCRU were included for completeness.

### Population and prevalence inputs

The Propeller target population for this model included patients aged ≥ 40 years with COPD. Patients were stratified by age category within the commercial and Medicare plans (i.e., 40–64 years and ≥ 65 years; patients aged < 40 years were excluded). Age distributions were obtained from the US Census Bureau in 2018 (Table 1) [19]. Prevalence of COPD was provided by age group (40–64 and ≥ 65 years) and estimated from the Centers for Disease Control and Prevention Behavioral Risk Factor Surveillance System survey [20]. COPD patients were grouped according to the GOLD 2017 ABCD assessment tool because this is a clinically relevant categorization that incorporates symptom burden and risk of exacerbation (based on prior exacerbation history). The proportion of patients within each the GOLD 2017 ABCD category was derived from Cabrera-Lopez et al. [21]. Prevalence and COPD group distributions are provided in Table 1. For the base case scenario, the estimated market penetration of Propeller adoption was 10% in year 1, 15% in year 2, and 30% in year 3.

### Medical healthcare resource use inputs

Baseline HCRU inputs were obtained from two published studies: Wallace et al. [5] and Bhatta [22]. Wallace et al. was a retrospective observational cohort study that quantified HCRU and costs according to COPD severity in both a commercially insured and Medicare population using GOLD stage 1–4 severity of airflow limitation criteria [5]. HCRU categories included office/outpatient visits, ED visits, hospitalizations and readmissions. Office and outpatient visits were combined into a single category, and readmissions were estimated by taking the proportion of hospitalizations that were readmissions (see Additional file 1, Section 1.1 for full details).

Unit cost data were based on Wallace et al. [5] by taking the total costs divided by the total units of HCRU reported in the study. All costs were calculated for each payer type and inflated to 2020 USD using the medical component of the consumer price index [23]. Wallace study data were derived from a commercial population and therefore unit costs were adjusted for a Medicare payer population to reflect lower unit costs. The commercial-to-Medicare payment ratios used were 2.64, 1.43, and 1.89 for hospital outpatient/ED, physician office and inpatient settings [24]. Calculated unit costs are provided in Table 1.

### Inhaler medication use and associated unit cost inputs

Mean baseline SABA use was obtained from Gondalia et al., who reported mean daily rescue inhaler use by COPD Assessment Test (CAT) score category (CAT 0–9, CAT 10–20, CAT 21–30 and CAT > 30) [7]. Baseline SABA use was used as a surrogate for symptoms and to quantify symptom-related costs (see Additional file 1, section 1.2 for full details) (Table 1). It was assumed that all patients with COPD included in our base case analysis are using a controller medication. Controller medications for COPD were any of the following: LAMA monotherapy; LABA monotherapy; LABA + ICS; LABA + LAMA; and LABA + LAMA + ICS.

The percentage distribution for each was allowed to differ according to GOLD 2017 ABCD category and was based on internal expert opinion (Table 1). The cost for each category was calculated by taking a simple average of the wholesale acquisition cost (WAC) prices [25] for available controller medications within each category divided by the total number of daily actuations based on labeled dosages.

Daily utilization and costs were calculated and weighted according to medication persistence, defined using the proportion of days covered (PDC) reported from Wallace et al. [5] (Table 1). To our knowledge, PDC according to GOLD 2017 ABCD category has not been estimated in the US. Estimates for PDC by GOLD category 1–4 category from Wallace 2019 were mapped to GOLD 2017 ABCD categories using data from Cabrera-Lopez et al. [21].

### Remote therapeutic monitoring

It was assumed that remote therapeutic monitoring (RTM) fees were paid at cost to the provider and that each provider billed for services using remote care current procedural terminology (CPT) codes. In the base case, we conservatively assumed all patients in commercial plans were eligible to have a claim submitted for reimbursement to their respective payer. Values were obtained from fee schedules used by the CMS for RTM. Assumed frequencies and fees are provided in Table 1.

### Effect of Propeller on medical HCRU

The base case assumed percentage reductions for medical HCRU and were calculated based on data from Alshabani et al. (see Additional file 1, section 1.3 for full details) [14]. Alshabani et al. conducted a retrospective analysis of patients in a quality improvement project in which changes in HCRU were assessed in pre- and post-electronic inhaler monitoring. Since the goal was to better understand the impact of the Propeller platform on subsequent HCRU, and due to a lack of published data on adherence in COPD patients using Propeller, controller medication costs related to potential increases in persistence were excluded from the base case analysis. In alternative scenarios, we also explored the relationship between hypothetical improvements in controller medication persistence on HCRU using a study by Toy et al. [26] rather than Alshabani et al. [14].

### Effect of Propeller on rescue and controller medications

The percent reduction in reliever medication use with Propeller was based on a pre-post observational study by Chen et al. [13]. This study enrolled 190 Medicare-eligible patients using a SABA medication with a physician-confirmed diagnosis of COPD and measured the change in daily SABA use after adopting Propeller. The reduction in SABA use from baseline to 12-month follow-up was used to calculate the percent reduction in SABA use resulting from the program. The model assumed no effect of Propeller on controller medication use due to an absence of data.

### Scenario and sensitivity analysis

The base case assumed inpatient and ED visit reductions from direct evidence by Alshabani et al. [14]. Under this scenario, changes in controller medication costs due to changes in adherence were not explicitly modeled. Alternative Scenario 1 assumed that the PDC for controller medications was increased by 0.10, and reductions in inpatient care and ED visits were 5.20% and 1.15%, respectively, based on Toy et al. [26]. Changes in controller medication costs due to changes in refill persistence were either included (1A) or excluded (1B). Alternative Scenario 2 assumed that the proportion of days covered for controller medications was increased by 0.30, and inpatient and ED visits were reduced by 15.60% and 3.45%, respectively, based on Toy et al. [26] Changes in controller medication costs due to changes in refill persistence were either included (2A) or excluded (2B).

For both commercially insured and Medicare populations, we conducted one-way sensitivity analyses on the base case medical resource unit costs (hospitalizations, ED visits), reductions in HCRU with Propeller, and reductions in SABA use with Propeller presented in Table 1, varying each input by −/+ 10% and −/+ 25%. Finally, we conducted a break-even analysis using the risk reduction in hospitalizations to understand the point at which the model results shift from cost savings to cost increases.

## Results

### Base case results

The number of annual eligible COPD patients for commercial and Medicare plan sizes of 5 million each was 212,200 and 606,600, respectively. Use of Propeller was projected to decrease total costs by $2,475 per enrolled patient [using the Propeller platform] for commercial payers and by $915 per enrolled patient for Medicare (Table 2). These reductions were primarily driven by lower estimated hospitalization costs for enrolled versus unenrolled patients [not using the Propeller platform] (differences of –$2975 and –$1574 from the commercial and Medicare perspectives, respectively). Smaller cost reductions were observed in the Medicare setting due to lower unit costs (reflecting lower reimbursement rates for Medicare versus commercial payers). The average cost per patient decreased with use of Propeller for all GOLD 2017 ABCD groups, and the magnitude of the reduction increased from GOLD stage A through D for both commercial (Additional file 1: Table S1) and Medicare (Additional file 1: Table S2) payers.

**Table 2:** Yearly cost per patient by enrollment status for commercial and Medicare payers

Costs, USD	Unenrolled	Enrolled	Difference
Commercial			
Total	14,048	11,573	− 2475
Propeller	0	200	200
Hospitalizations	9918	6943	− 2975
ED Visits	462	208	− 254
Outpatient/physician office	333	333	0
Remote monitoring	0	655	655
SABA use	292	119	− 174
Controller medication use	3042	3042	0
Medicare			
Total	8990	8075	− 915
Propeller	0	200	200
Hospitalizations	5248	3673	− 1574
ED Visits	175	79	− 96
Outpatient/physician office	233	233	0
Remote monitoring	0	655	655
SABA use	292	119	− 174
Controller medication use	3042	3042	0

ED: emergency department; SABA: short-acting beta-agonist

Based on hypothetical estimated market penetration assumptions, the total number of patients projected to use Propeller in years 1, 2, and 3 was 21,220, 31,830 and 63,660 respectively, from a commercial payer perspective, and 60,660, 90,990 and 181,980, respectively, from a Medicare payer perspective.

From a commercial payer perspective, annual expenditures for Propeller and provider RTM CPT claims were projected to increase by $4.2 million ($0.07 per-member per-month [PMPM]) and $13.9 million ($0.23 PMPM), respectively in year 1, to $12.7 million ($0.21 PMPM) and $41.7 million ($0.69 PMPM) in year 3 (Table 3). After accounting for projected reductions in hospitalizations, ED visits and SABA use, total average savings of approximately $288.8 million (–$1.60 PMPM) were projected (Table 3).

**Table 3:** Per-member per-month budget impact estimates from a commercial payer perspective

Costs, USD	Year 1	Year 2	Year 3	Average
PMPM budget impact				
Total	− 0.88	− 1.31	− 2.63	− 1.60
Propeller	0.07	0.11	0.21	0.13
Hospitalizations	− 1.05	− 1.58	− 3.16	− 1.93
ED Visits	− 0.09	− 0.13	− 0.27	− 0.16
Outpatient/physician office	0.00	0.00	0.00	0.00
Remote monitoring	0.23	0.35	0.69	0.42
SABA use	− 0.06	− 0.09	− 0.18	− 0.11
Controller medication use	0.00	0.00	0.00	0.00
PMPM costs without propeller				
Total	49.68	49.68	49.68	49.68
Propeller	0.00	0.00	0.00	0.00
Hospitalizations	35.08	35.08	35.08	35.08
ED Visits	1.63	1.63	1.63	1.63
Outpatient/physician office	1.18	1.18	1.18	1.18
Remote monitoring	0.00	0.00	0.00	0.00
SABA use	1.03	1.03	1.03	1.03
Controller medication use	10.76	10.76	10.76	10.76
PMPM costs with Propeller				
Total	48.81	48.37	47.06	48.08
Propeller	0.07	0.11	0.21	0.13
Hospitalizations	34.02	33.50	31.92	33.15
ED Visits	1.54	1.50	1.36	1.47
Outpatient/physician office	1.18	1.18	1.18	1.18
Remote monitoring	0.23	0.35	0.69	0.42
SABA use	0.97	0.94	0.85	0.92
Controller medication use	10.76	10.76	10.76	10.76

ED: emergency department; PMPM: per-member per-month; SABA: short-acting beta-agonist

For Medicare, Propeller and provider RTM CPT claims expenditures were estimated to increase by $12.1 million ($0.20 PMPM) and $39.7 million ($0.66 PMPM) in year 1, and by $36.4 million ($0.61 PMPM) and $119.2 million ($1.99) PMPM in year 3 (Table 4). Total average savings of $305.6 million (–$1.70 PMPM) were estimated based on savings from reduced hospitalizations, ED visits, and SABA use (Table 4).

**Table 4:** Per-member per-month budget impact estimates from a Medicare payer perspective

Costs, USD	Year 1	Year 2	Year 3	Average
PMPM budget impact				
Total	− 0.93	− 1.39	− 2.78	− 1.70
Propeller	0.20	0.30	0.61	0.37
Hospitalizations	− 1.59	− 2.39	− 4.77	− 2.92
ED Visits	− 0.10	− 0.15	− 0.29	− 0.18
Outpatient/physician office	0.00	0.00	0.00	0.00
Remote monitoring	0.66	0.99	1.99	1.21
SABA use	− 0.18	− 0.26	− 0.53	− 0.32
Controller medication use	0.00	0.00	0.00	0.00
PMPM costs without propeller				
Total	90.89	90.89	90.89	90.89
Propeller	0.00	0.00	0.00	0.00
Hospitalizations	53.05	53.05	53.05	53.05
ED Visits	1.77	1.77	1.77	1.77
Outpatient/physician office	2.36	2.36	2.36	2.36
Remote monitoring	0.00	0.00	0.00	0.00
SABA use	2.96	2.96	2.96	2.96
Controller medication use	30.76	30.76	30.76	30.76
PMPM costs with propeller				
Total	89.97	89.50	88.12	89.20
Propeller	0.20	0.30	0.61	0.37
Hospitalizations	51.46	50.67	48.28	50.14
ED Visits	1.67	1.62	1.48	1.59
Outpatient/physician office	2.36	2.36	2.36	2.36
Remote monitoring	0.66	0.99	1.99	1.21
SABA use	2.78	2.69	2.43	2.64
Controller medication use	30.76	30.76	30.76	30.76

ED: emergency department; PMPM: per-member per-month; SABA: short-acting beta-agonist

### Scenario analyses

#### Commercially insured patients

Under Alternative Scenario 1 (assuming a 10% increase in adherence), the estimated 3-year budget impact was an increase in PMPM cost of $0.49 and $0.14 when controller medication use was included and excluded, respectively (Table 5). Under alternative scenario 2 (assuming a 30% increase in adherence), the 3-year budget impact was estimated to be $0.50 and –$0.55 PMPM when controller medications were included and excluded, indicating net savings when increases in medication adherence were not considered (Table 5). When excluding increases in costs due to controller medications, an increase in PDC of 14.16 percentage points was estimated to result in budget neutrality in the commercial patient payer population.

**Table 5:** Per-member per-month 3-year budget impact results from scenario analyses

Costs, USD	Base case	Scenario 1A	Scenario 1B	Scenario 2A	Scenario 2B
Commercial perspective					
Budget impact	− 1.60	0.49	0.14	0.50	− 0.55
Propeller	0.13	0.13	0.13	0.13	0.13
Hospitalizations	− 1.37	− 0.33	− 0.33	− 1.00	− 1.00
ED visits	− 0.16	− 0.01	− 0.01	− 0.03	− 0.03
Outpatient/physician office	0.00	0.00	0.00	0.00	0.00
Remote monitoring	0.42	0.42	0.42	0.42	0.42
SABA use	− 0.11	− 0.11	− 0.11	− 0.11	− 0.11
Controller medication use	0.00	0.35	Not included	1.04	Not included
Medicare perspective					
Budget impact	− 1.70	1.88	0.88	2.83	− 0.15
Propeller	0.37	0.37	0.37	0.37	0.37
Hospitalizations	− 2.92	− 0.51	− 0.51	–1.52	− 1.52
ED Visits	− 0.18	− 0.01	− 0.01	− 0.03	− 0.03
Outpatient/physician office	0.00	0.00	0.00	0.00	0.00
Remote monitoring	1.21	1.21	1.21	1.21	1.21
SABA use	− 0.32	− 0.32	− 0.32	− 0.32	− 0.32
Controller medication use	0.00	0.99	Not included	2.98	Not included

ED: emergency department; PMPM: per-member per-month; SABA: short-acting beta-agonist

Alternative Scenario 1: PDC for controller medications increased by 0.10 and reductions in inpatient care (5.20%) and ED visits (1.15%) where changes in controller medication costs due to changes in refill persistence either included (1A) or excluded (1B)

Alternative Scenario 2: PDC for controller medications was increased by 0.30 and reductions in inpatient care (15.60%) and ED visits (3.45%) where changes in controller medication costs due to changes in refill persistence either included (2A) or excluded (2B)

Under Alternative Scenario 1A, adoption of Propeller across all GOLD 2017 ABCD categories was estimated to result in a net cost increase; however, when controller medication costs were excluded (Alternative Scenario 1B), net savings was projected for GOLD D (Additional file 1: Table S1). Under Alternative Scenario 2, Propeller was estimated to result in a net savings across all groups (Alternative Scenario 2B), except GOLD A and B when controller medication costs were included (Alternative Scenario 2A) (Additional file 1: Table S1).

### Medicare insured patients

Under Alternative Scenario 1, the 3-year budget impact was $1.88 and $0.88 PMPM with and without controller medication costs, respectively (Table 5). Under Alternative Scenario 2, the budget impact was estimated to be $2.83 and $–0.15 PMPM with and without controller medication costs, respectively (Table 5). When increases in controller medication costs were excluded, budget neutrality was estimated when PDC increased by 27.03 percentage points. Under Alternative Scenario 1, all GOLD categories were projected to result in a net increase in costs, regardless of whether or not controller medication costs were included (Additional file 1: Table S2). Under Alternative Scenario 2, Propeller was projected to result in a net savings for GOLD B, GOLD C and GOLD D only when controller medication costs were excluded (Alternative Scenario 2B) (Additional file 1: Table S2).

### Sensitivity analyses

#### Commercially insured patients

In a commercial payer plan, by varying either medical resource unit costs or reductions in HCRU with Propeller by −/+ 10%, use of Propeller was projected to decrease total costs per enrolled patient per year by $2152 to $2797 and varying either of these parameters by −/+ 25% was projected to decrease total costs per enrolled patient per year by $1668 to $3282. By varying the assumed base case reduction in SABA use with Propeller (59.4%) by −/+ 10% and −/+ 25%, use of Propeller was projected to decrease total costs per enrolled patient per year by $2457 to $2492 and $2431 to $2518, respectively.

The base case model assumed a percentage reduction of 30% for hospitalizations. In the break-even analysis, a percentage reduction of 5.04855% in hospitalizations would result in equivalent per annual per-patient costs for commercial plan patients enrolled and not enrolled in Propeller.

#### Medicare insured patients

In a Medicare plan, by varying either medical resource unit costs or reductions in HCRU with Propeller, Propeller was projected to decrease total costs per enrolled patient per year by $749 to $1083 (−/+10%) and by $499 to $1333 (−/+25%). By varying the assumed base case reduction in SABA use with Propeller, Propeller was projected to decrease total costs per enrolled patient per year by $898 to $933 (−/+10%) and $872 to $959 (−/+25%).

The base case model assumed a percentage reduction of 30% for hospitalizations. In the break-even analysis, a percentage reduction of 12.547% in hospitalizations would result in equivalent per annual per-patient costs for Medicare patients enrolled and not enrolled in Propeller.

## Discussion

This analysis projected costs savings with the use of Propeller for patients with COPD. The estimated cost savings were directly driven by assumptions about HCRU during use of Propeller (i.e., primarily driven by reductions in ED visits and hospitalizations, and to a lesser extent by SABA use due to the low cost of these medications). Assumptions that HCRU would decrease with Propeller were based on direct evidence showing decreases in each of the HCRU parameters after initiation of Propeller [13, 14]. Data from these sources (Chen et al. [13] and Alshabani et al. [14] were obtained from pre-post observations rather than comparative analyses and are therefore subject to uncertainty in the true effect of Propeller on each HCRU type. For example, the Chen et al. study was limited by its focus on rescue medication rather than controller medication, and possible use of additional non-censored rescue medication included nebulizers [13]. Nonetheless, higher SABA use has demonstrated a clinically meaningful association with higher GOLD stages (these groups are at greater risk of COPD exacerbations) [7, 12] and with periods of moderate-to-severe exacerbations, where further clinical intervention may be performed to reduce the risk of costly hospitalizations [10, 11]. We also tested assumptions around HCRU reductions in our scenario analysis, which showed that savings are highest for COPD patients meeting GOLD 2017 C and D criteria (i.e. those with recurrent exacerbations).

COPD patients using Propeller have demonstrated high levels of adherence to controller medications that increase with older age categories. While evidence exists for improvements in adherence among asthma patients managed with Propeller [9, 27, 28], no studies have directly evaluated the effect of Propeller on adherence in patients with COPD. Although no studies have directly evaluated the impact of Propeller on adherence among patients with COPD, patients with COPD and asthma have been shown to have similar adherence to controller medications while using Propeller [29].

The goal of the analysis was to estimate the budget impact of the Propeller platform in COPD vis-à-vis downstream healthcare resource use reductions that have been previously demonstrated in the published literature [13, 14]. As such, we included the price for Propeller, remote monitoring payments, and downstream healthcare resource use and excluded increased costs due to increased medication adherence in the base case. Although the base case assumed no effect on increased adherence to controller medications, we evaluated increased adherence in our scenario analysis. The scenario analysis estimated the effect of hypothetical increases in persistence on the budget impact and costs. The use of these alternative scenarios was considered to be conservative, given that only modest decreases in HCRU were estimated under these scenarios and would not include other benefits of Propeller unrelated to medication adherence, because the platform also allows for closer monitoring of medication use behavior and earlier identification of patients who are increasingly symptomatic and may require treatment modifications. COPD patients meeting GOLD C and D criteria may benefit clinically from increased adherence to controller medications as well as resulting in cost savings even when the cost of these medications are taken into consideration.

Most studies evaluating the effects of Propeller on HCRU or outcomes provided ongoing care management as part of the intervention. No studies have been published demonstrating effects of Propeller on COPD-related HCRU in the absence of concomitant care management. It is unknown to what extent care management contributes to overall effects and/or whether care management without Propeller would result in similar outcomes. Although care management may be optional with Propeller, studies have shown that engaging patients with structured self-management for COPD can also have an effect on improving outcomes, including reduction of HCRU [30]. In the absence of concomitant HCP care management, Propeller may be a useful tool to aid in self-management and monitoring of symptoms [8].

In this analysis, the base case analysis only included COPD-related HCRU reductions. Patients with COPD have a high prevalence of comorbidities, including cardiovascular disease and diabetes, and these comorbidities contribute to increases in COPD-related exacerbations and mortality. Conversely, an interplay between COPD and comorbidities has been suggested, indicating that controlling COPD disease activity may help reduce the burden related to comorbid conditions [31].

A key limitation at the time of this analysis was the limited availability of published literature, which may have influenced the model base case, scenario and sensitivity analysis results and therefore the conclusions of this study. Most studies used for key inputs were small and focused on a single outcome, therefore we had to use multiple sources for the base case inputs. The pre-post single arm designs of these studies also increase uncertainty around the true impact of Propeller on HCRU, due to the lack of a control group and the possibility of regression to the mean with repeated measures. For example, the results of our break-even analysis are subject to uncertainty in the effect size of Propeller on hospitalizations, particularly given the enrollment of individuals with high baseline utilization [14]. Without a control arm in Chen et al., it is difficult to estimate the natural course of SABA use over time, and whether the observed results over- or underestimate the effect of Propeller [13]. This is especially true given the progressive nature of COPD, and the fact that SABA use depends on many factors, including whether a patient is newly diagnosed, disease stage, COPD exacerbations, and use of maintenance medications [32].

Estimated results may not be generalizable to all real-world settings. Although baseline HCRU and inhaler utilization data are largely based on the published literature, actual HCRU for COPD in commercial and Medicare payers may be substantially different due to differences in standards of care and other types of interventions that impact each patient population. For this budget impact model, we did not have access to data (e.g., administrative claims data) to calculate weighted average for WAC prices. Actual prices paid for prescription medications may vary from what has been included in our study. Furthermore, the current findings are only applicable to the specific settings in which costs were estimated and may not apply to other health care settings or health systems.

## Conclusion

Our analysis suggests a potentially substantial reduction in COPD-related costs for commercial and Medicare payers with use of Propeller, largely due to estimated decreases in hospitalizations and ED visits. Although increases in adherence to controller medications in COPD patients may offset cost savings, improved adherence may represent an important outcome of interest to patients, HCPs and payers. Further study is required to validate HCRU reductions in comparative effectiveness studies with standard of care for COPD, allowing for updated budget impact scenario analyses to be evaluated.

## Supplementary Information

**Additional file 1**. Supplementary Methods: Section 1.1 Medical Healthcare Resource Use Inputs; Section 1.2 Inhaler Medication Use and Associated Unit Cost Inputs; Section 1.3 Effect of Propeller on Medical HCRU; **Table S1**. Annual per-patient estimated cost by enrolment status and severity for commercial payers; **Table S2**. Annual per-patient estimated cost by enrolment status and severity for Medicare.

## Data Availability

All data generated by or used in the analyses are included in the published article and its supplementary information files.

## Abbreviations

CAT: COPD Assessment Test
CMS: Centers for Medicare & Medicaid Services
COPD: Chronic obstructive pulmonary disease
CPT: Current procedural terminology
ED: Emergency department
GOLD: Global Initiative for Chronic Obstructive Lung Disease
HCP: Healthcare provider
HCRU: Healthcare resource utilization
ICS: Inhaled corticosteroid
LABA: Long-acting beta-agonist
LAMA: Long-acting muscarinic antagonist
PDC: Proportion of days covered
PMPM: Per-member per-month
RTM: Remote therapeutic monitoring
SABA: Short-acting beta-agonist
US: United States
WAC: Wholesale acquisition cost
