# Supplementary material for: Budget impact analysis of a digital monitoring platform for COPD
Source: Cost Eff Resour Alloc. 2023 Jun 4;21:36. doi: 10.1186/s12962-023-00443-x (PMC10240705; doi:10.1186/s12962-023-00443-x)
Supplement: Supplementary file 1 — Additional file 1. Supplementary Methods: Section 1.1 Medical Healthcare Resource Use Inputs; Section 1.2 Inhaler Medication Use and Associated Unit Cost Inputs; Section 1.3 Effect of Propeller on Medical HCRU; Table S1. Annual per-patient estimated cost by enrolment status and severity for commercial payers; Table S2. Annual per-patient estimated cost by enrolment status and severity for Medicare. [file 12962_2023_443_MOESM1_ESM.docx]

**Additional File 1**

**Budget impact analysis of a digital monitoring platform for COPD**

Tim J Inocencio PhD,^1^ Kimberly L Sterling PharmD,^2^ Sibel Sayiner MS,^3^ Michael E Minshall MPH,^2^ Leanne Kaye PhD,^2^ Umur Hatipoğlu MD^4^

^1^OPEN Health Evidence and Access, Parsippany, NJ, USA; ^2^ResMed Science Center, San Diego, CA, USA; ^3^Propeller Health, San Francisco, CA, USA; ^4^Cleveland Clinic, Cleveland, OH, USA

**1. SUPPLEMENTARY METHODS**

**1.1 Medical Healthcare Resource Use Inputs**

To our knowledge, data quantifying healthcare resource use (HCRU) according to 2017 GOLD ABCD groups in the US have not been produced. Because HCRU was given by GOLD stage 1–4 rather than GOLD ABCD groups, it was assumed that patients in GOLD stage 1 and GOLD A had equivalent HCRU. These categories include a small number of patients and both have low HCRU. Data from the HUNT study (Trøndelag Health study) were used to estimate HCRU for patients with GOLD B–D COPD relative to GOLD A.(1) The HUNT study included 1300 participants with COPD aged ≥40 years in Norway identified from 1995 through 1997 and followed until December 31, 2015. Hospitalization rates were evaluated in accordance with GOLD 2007, GOLD 2011 and GOLD 2017 criteria. To align with more contemporary definitions of COPD burden, we used HCRU estimates using the GOLD 2017 criteria. Because of small sample sizes in both the GOLD C and GOLD D groups, we assumed that HCRU for GOLD C patients was equal to that for GOLD D patients. The ratio for HCRU in GOLD B and D to GOLD A in the HUNT study was then applied to the GOLD estimate obtained through the Wallace study (as described above) to obtain US-specific COPD-related HCRU for patients with GOLD A and GOLD B COPD.

**1.2 Inhaler Medication Use and Associated Unit Cost Inputs**

To assign mean puffs according to the GOLD ABCD criteria used in the model, baseline SABA use for CAT scores 0–9 was assumed for GOLD A and GOLD C categories. Baseline SABA use for CAT scores 10–20 was assumed for GOLD B, while the average of baseline SABA use for CAT scores 21–30 and >30 was assumed for GOLD D. The cost per SABA actuation was calculated by dividing the wholesale acquisition cost (WAC) by the total number of actuations for each SABA medication and taking the average of all products.

**1.3 Effect of Propeller on Medical HCRU**

Alshabani *et al* (2020) was a 39-patient retrospective observational study compared annual HCRU before and after initiation of the Propeller intervention, and found a statistically significant decrease in COPD-related hospitalizations and ED visits after initiating Propeller.(2) The percentage reductions for COPD-related hospitalizations and ED visits were calculated based on the mean annual COPD-related hospitalizations and ED visits reported, and applied according to the estimates of baseline HCRU estimated from the Wallace 2019 study.(3) Since no data for office/outpatient visits were available, 0% was assumed (i.e. no effect). The calculated percent reductions for HCRU and ED visits when Propeller was used are provided in Table 1.

The study by Toy *et al* (2011) reported the relationship between refill persistence (estimated using the proportion of days covered) and downstream HCRU, including hospitalization and ED visits.(4) A 2.6% and 1.8% decrease in all-cause HCRU was estimated for every 5% increase in PDC. These estimates were then applied to the assumed baseline HCRU to obtain potential reductions in persistence-based HCRU across a range of scenarios by increasing PDC between 10% and 30% from the base case values.

**Table S1.** Annual per-patient estimated cost by enrollment status and severity for commercial payers

|  | **Total cost, USD** | | **Difference** |
| --- | --- | --- | --- |
|  | **Unenrolled** | **Enrolled** |  |
| **Base Case** |  |  |  |
| GOLD A  GOLD B  GOLD C  GOLD D | 10,97211,064  16,283  18,419  19,482 | 9,500  13,136  14,500  15,400 | –1,563  –3,147  –3,919  –4,082 |
| **Alternative Scenario 1A**  GOLD A  GOLD B  GOLD C  GOLD D | 11,06416,283  18,419  19,482 | 12,011  16,895  18,919  19,900 | 947  612  500  418 |
| **Alternative Scenario 1B**  GOLD A  GOLD B  GOLD C  GOLD D | 8,134  13,118  15,706  15,980 | 8,538  13,202  15,722  15,833 | 404  84  16  –147 |
| **Alternative Scenario 2A**  GOLD A  GOLD B  GOLD C  GOLD D | 11,06416,283  18,419  19,482 | 12,319  16,675  18,334  19,477 | 1,255  391  –85  –6 |
| **Alternative Scenario 2B**  GOLD A  GOLD B  GOLD C  GOLD D | 8,134  13,118  15,706  15,980 | 7,761  11,926  14,168  14,280 | –373  –1,192  –1,538  –1,700 |

**Abbreviations:** GOLD, Global initiative of Obstructive Lung Disease.

**Table S2.** Annual per-patient estimated cost by enrollment status and severity for Medicare

|  | **Total cost, USD** | | **Difference** |
| --- | --- | --- | --- |
|  | **Unenrolled** | **Enrolled** |  |
| **Base Case** |  |  |  |
| GOLD A  GOLD B  GOLD C  GOLD D | 7,347  10,244  11,087  12,150 | 6,921  8,964  9,442  10,342 | –426  –1,279  –1,645  –1,808 |
| **Alternative Scenario 1A**  GOLD A  GOLD B  GOLD C  GOLD D | 7,347  10,244  11,087  12,150 | 8,479  11,159  11,956  12,937 | 1,131  915  869  787 |
| **Alternative Scenario 1B**  GOLD A  GOLD B  GOLD C  GOLD D | 4,417  7,078  8,373  8,647 | 5,006  7,466  8,758  8,870 | 589  388  385  222 |
| **Alternative Scenario 2A**  GOLD A  GOLD B  GOLD C  GOLD D | 4,4177,347  10,244  11,087  12,150 | 9,157  11,545  12,109  13,252 | 1,809  1,301  1,023  1,102 |
| **Alternative Scenario 2B**  GOLD A  GOLD B  GOLD C  GOLD D | 4,417  7,078  8,373  8,647 | 4,599  6,796  7,944  8,055 | 182  –281  –429  –592 |

**Abbreviations:** GOLD, Global initiative of Obstructive Lung Disease.

**REFERENCES**

1. Bhatta L, Leivseth L, Mai XM, Henriksen AH, Carslake D, Chen Y, et al. GOLD classifications, COPD hospitalization, and all-cause mortality in chronic obstructive pulmonary disease: the HUNT study. Int J Chron Obstruct Pulmon Dis. 2020;15:225-33.

2. Alshabani K, Attaway AA, Smith MJ, Majumdar U, Rice R, Han X, et al. Electronic inhaler monitoring and healthcare utilization in chronic obstructive pulmonary disease. J Telemed Telecare. 2020;26(7-8):495-503.

3. Wallace AE, Kaila S, Bayer V, Shaikh A, Shinde MU, Willey VJ, et al. Health care resource utilization and exacerbation rates in patients with COPD stratified by disease severity in a commercially insured population. J Manag Care Spec Pharm. 2019;25(2):205-17.

4. Toy EL, Beaulieu NU, McHale JM, Welland TR, Plauschinat CA, Swensen A, et al. Treatment of COPD: relationships between daily dosing frequency, adherence, resource use, and costs. Respir Med. 2011;105(3):435-41.
